# Systematic Review and Meta-Analysis of the Blood Glutathione Redox State in Chronic Obstructive Pulmonary Disease

**DOI:** 10.3390/antiox9111146

**Published:** 2020-11-18

**Authors:** Salvatore Sotgia, Panagiotis Paliogiannis, Elisabetta Sotgiu, Sabrina Mellino, Elisabetta Zinellu, Alessandro G. Fois, Pietro Pirina, Ciriaco Carru, Arduino A. Mangoni, Angelo Zinellu

**Affiliations:** 1 Department of Biomedical Sciences, School of Medicine, University of Sassari, 07100 Sassari, Italy; eli.sotgiu@alice.it (E.S.); sabrinamellino3@gmail.com (S.M.); carru@uniss.it (C.C.); azinellu@uniss.it (A.Z.); 2 Department of Clinical and Experimental Medicine, School of Medicine, University of Sassari, 07100 Sassari, Italy; panospaliogiannis@gmail.com (P.P.); agfois@uniss.it (A.G.F.); pirina@uniss.it (P.P.); 3 Department of Respiratory Diseases, University Hospital Sassari (AOU-SS), 07100 Sassari, Italy; elisabetta.zinellu@aousassari.it; 4 Quality Control Unit, University Hospital Sassari (AOU-SS), 07100 Sassari, Italy; 5 Discipline of Clinical Pharmacology, College of Medicine and Public Health, Flinders University and Flinders Medical Centre, Adelaide 5001, Australia; arduino.mangoni@flinders.edu.au

**Keywords:** chronic obstructive pulmonary disease (COPD), glutathione, oxidative stress

## Abstract

The aim of this systematic review and meta-analysis was to assess the blood concentrations of the total and reduced forms of the low-molecular-weight antioxidant thiol glutathione (GSH) in chronic obstructive pulmonary disease (COPD) patients in comparison to healthy individuals. A literature search was conducted in the PubMed and Web of Science databases from inception until June 2020. In the 18 studies identified (involving a total of 974 COPD patients and 631 healthy controls), the pooled reduced GSH concentrations were significantly lower in patients with COPD than controls (SMD  =  −3.04, 95% CI = −4.42 to −1.67; *p*  <  0.001). By contrast, the pooled total GSH concentrations were significantly higher in patients with COPD than controls (SMD = 0.42, 95% CI = 0.11 to 0.73; *p* = 0.009). Our meta-analysis showed that the blood concentrations of reduced GSH, even in the presence of higher total GSH concentrations, were significantly lower in patients with COPD when compared to healthy controls. This suggests that an impaired antioxidant defense system plays an important role in the pathogenesis of COPD.

## 1. Introduction

Chronic obstructive pulmonary disease (COPD), a non-communicable respiratory disease with an estimated prevalence of 4–20% in adults over 40 years worldwide, is characterized by progressive airflow limitation and emphysematous changes in the lungs [1,2]. According to the Global Burden of Disease Study, COPD is the third leading cause of death worldwide and is expected to become the leading cause by the year 2030 [3]. COPD is typically characterized by a chronic local and systemic inflammatory response that is largely driven by oxidative stress [4,5]. The increased oxidative burden in the lungs is due to exposure to environmental oxidants, such as air pollution, occupational dust or cigarette smoking [6,7], as well as the endogenous generation of oxidants by several types of cells and the mitochondrial electron transport chain [4]. Oxidative stress, resulting from an excess of oxidants and/or a deficit in antioxidant defense mechanisms, favors lung tissue damage and activates specific molecular mechanisms that initiate local inflammation [8]. The latter involves the activation of epithelial cells and resident macrophages, and the recruitment and activation of neutrophils, eosinophils, monocytes and lymphocytes that release a variety of pro-inflammatory mediators, including cytokines, chemokines, growth factors and lipid mediators [4]. Both inflammatory and structural cells also produce reactive oxygen species (ROS) [9,10], with a consequent shift in the intracellular thiol/disulfide redox state toward more oxidative conditions [11]. The perturbation of thiol concentrations in COPD is therefore regarded as the hallmark of oxidative stress, which involves either excessive ROS production and/or impaired antioxidant defense mechanisms [12]. Among the low-molecular-weight thiols, glutathione (GSH, l-γ-glutamyl-l-cysteinyl-glycine) accounts for 90% of intracellular non-protein thiols [13]. The assessment of the concentrations of GSH, one of the primary non-enzymatic antioxidants in the lung, has received significant attention in COPD [14], as it plays a key role in the maintenance of cellular redox potentials and homeostatic mechanisms [15]. GSH concentrations have been investigated in different biological matrices such as sputum [16], exhaled breath condensate [17], bronchoalveolar lavage [18], blood [19] and lung tissue [20]. The concentrations of GSH in biological fluids that are functionally and anatomically close to the lung are several times higher than those in the plasma, e.g., 200–400 µM in the epithelial lining fluid vs. 2–4 µM in the plasma [21]. However, the collection of lung lining specimens is invasive and not well standardized [22], as there is no consensus on how to normalize the dilution process introduced during the sampling. Therefore, albeit with the inherent limitation of not capturing the local antioxidant/oxidant balance, studies in COPD have generally focused on circulating GSH concentrations, with conflicting results. We sought to address these discrepancies by conducting a systematic review and meta-analysis on the available evidence regarding the association between blood GSH concentrations and COPD.

## 2. Materials and Methods

### 2.1. Search Strategy, Eligibility Criteria and Study Selection

An electronic search was performed in the PubMed and Web of Science databases, from inception until June 2020, using a combination of the following terms: glutathione, GSH, chronic obstructive pulmonary disease, and COPD. The references of the retrieved articles were also searched to identify additional studies. The full-text paper selection criteria included (i) primary research studies, (ii) assessments of GSH in red blood cells or whole blood, (iii) case–control designs, (iv) sample sizes ≥10 patients with COPD and (v) full-text papers in English. The articles were independently reviewed by two investigators. A third investigator was involved in the case of disagreement between peers. The quality of each study was assessed using the Newcastle–Ottawa scale [23], which takes into account the cohort selection, the comparability of the cohorts based on the design or analysis, how the exposure was determined and how the outcomes of interest were evaluated. A score of six or more was taken as the quality threshold.

### 2.2. Statistical Analysis

Because of the different units of measurement (g/L, mol/L or mol/g Hb) used to express the blood GSH concentrations in primary studies, forest plots of continuous data and the assessment of the differences in blood GSH concentrations in COPD patients vs. healthy controls were expressed using the standardized mean differences (SMDs). When primary studies reported concentrations as median and interquartile range (IQR), the values of the mean and standard deviation were estimated as described by Wan et al. (2014) [24]. When the standard error (SE) was used instead of the standard deviation, the latter was estimated by multiplying the SE by the square root of the sample size. The heterogeneity between studies was assessed with Cochran’s Q statistic, with a significance level of *p* < 0.10. The magnitude of the heterogeneity was assessed with the I^2^ statistic, and the I^2^ values were interpreted as follows: I^2^ < 25%, no heterogeneity; I^2^ between 25% and 50%, moderate heterogeneity; I^2^ between 50% and 75%, large heterogeneity; and I^2^ > 75%, extreme heterogeneity [25,26]. A random-effects model was used to perform the meta-analysis in the case of substantial heterogeneity. Sensitivity analysis was performed to assess the robustness of the meta-analysis, excluding a single case-control study each time and recalculating the effect size [27]. The presence of publication bias and the associations between the size of the study and the size of the effect were assessed, respectively, by Egger’s regression asymmetry test at a *p* < 0.05 level of significance and the Begg’s adjusted rank correlation [28,29]. The Duval and Tweedie “trim and fill” procedure was carried out to further test the occurrence of publication bias [30]. Confidence intervals at 95% (CIs) were reported for each effect size and the overall effect, and *p* < 0.05 was considered statistically significant. The study was fully compliant with the principles outlined in the PRISMA Statement [31]. Statistical analyses were performed using Small Stata for Windows, version 14.1, 64 bit (StataCorp LP, College Station, TX, USA).

## 3. Results

### 3.1. Literature Search and Study Selection

**Figure 1:**
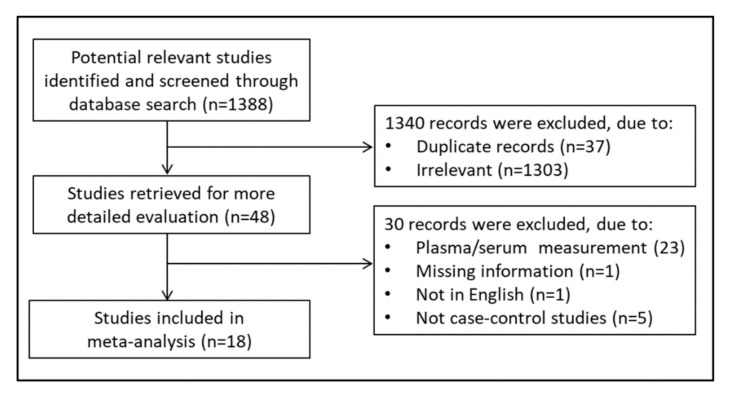
Flow chart of study selection.

A flow chart describing the screening process is presented in Figure 1.

The initial search yielded 1388 articles. Of them, 1340 were excluded because they were either irrelevant or duplicates. After a full-text review of the remaining 48 articles, a further 30 were excluded, as they did not meet the inclusion criteria, leaving the remaining 18 for further analysis [32,33,34,35,36,37,38,39,40,41,42,43,44,45,46,47,48,49]. The retrieved studies were published between 2002 and 2019, and their characteristics are described in Table 1.

A total of 1605 subjects, 974 COPD patients (82% males) and 631 controls (72% males), were evaluated. The mean age ranged between 42.0 and 75.7 years in the COPD patients and between 43.7 and 77.7 years in the controls. The blood concentrations of reduced GSH (rGSH) were measured in 14 studies [34,35,36,37,39,40,41,42,44,45,46,47,48,49], while total GSH (tGSH) was measured in four studies [32,33,38,43]. GSH was measured in red blood cells (RBCs) in nine studies and in whole blood (WB) in the other nine. The GSH concentrations were reported as the median and IQR, mean and SE, and mean and standard deviation. COPD was diagnosed according to the GOLD guidelines in nine articles [33,37,38,42,43,44,45,46,48], the ATS/ERS guidelines in two studies [41,47] and the BTS guidelines in one study [34]. Six studies did not report the guidelines used for COPD diagnosis [32,35,36,39,40,49].

### 3.2. Blood Reduced GSH (rGSH) and COPD

**Figure 2:**
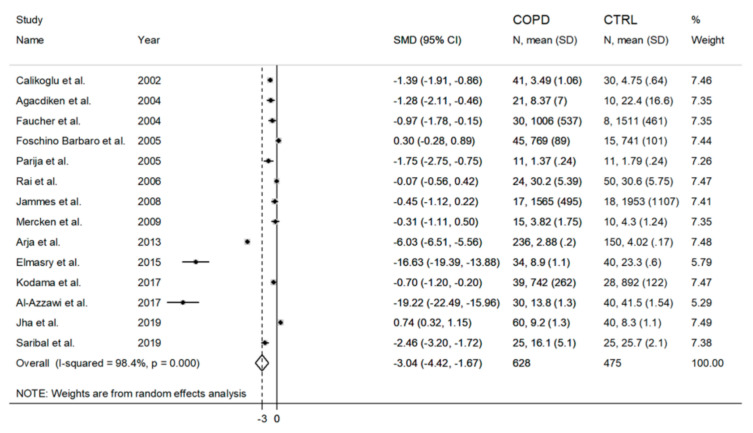
Forest plot of studies examining blood reduced GSH and COPD.

When compared to healthy controls, the blood (RBCs/WB) rGSH concentrations in COPD patients were significantly lower in 10 studies [34,35,36,39,41,44,45,46,47,49], significantly higher in one study [48], and non-significantly different in the remaining three studies [37,40,42]. A forest plot of the blood rGSH concentrations in COPD patients vs. healthy subjects is presented in Figure 2.

**Figure 3:**
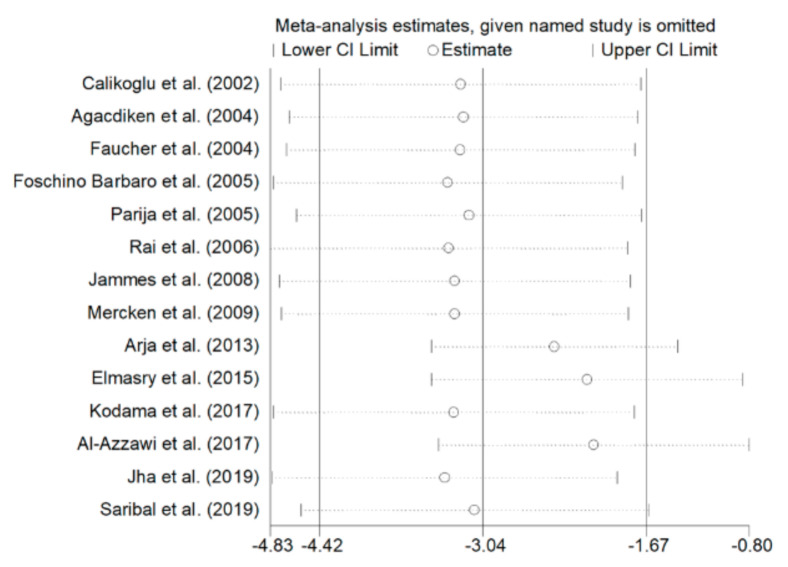
Sensitivity analysis of the association between blood reduced GSH and COPD. The influence of individual studies on the overall standardized mean difference (SMD) is shown. The middle vertical axis indicates the overall SMD, and the two vertical axes indicate the 95% confidence intervals (CI). Hollow circles represent the pooled SMD when the remaining study was omitted from the meta-analysis. Two ends of each broken line represent 95% CIs.

Because of the high heterogeneity (I^2^  =  98.4%, *p*  <  0.001), a random-effects model was used to calculate the pooled standardized mean difference (SMD). The latter showed that the rGSH concentrations were significantly lower in COPD patients than controls (SMD  =  −3.04, 95% CI = −4.42 to −1.67; *p*  <  0.001). Despite the potentially distortive effects of two studies [45,47], a sensitivity analysis performed by recalculating the confidence interval of the pooled effect size after eliminating each study at a time showed no substantial modification of the effect size, which ranged between −2.10 and −3.37 (Figure 3).

**Figure 4:**
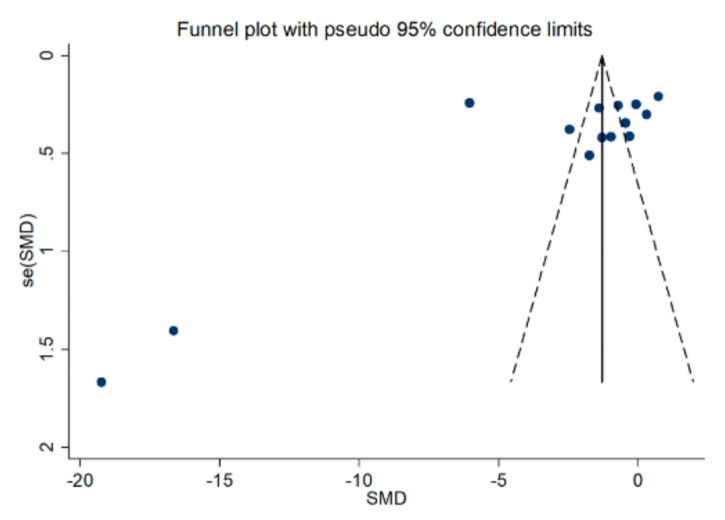
Funnel plot of studies investigating healthy controls and patients with COPD.

The funnel plot in Figure 4 confirms the distortive effects of the two above-mentioned studies. Their removal attenuated the effect size (SMD = −1.20, 95% CI = −2.42 to 0.030, *p* = 0.056) without influencing the magnitude of the heterogeneity (I^2^ = 98.0%, *p* < 0.001).

**Figure 5:**
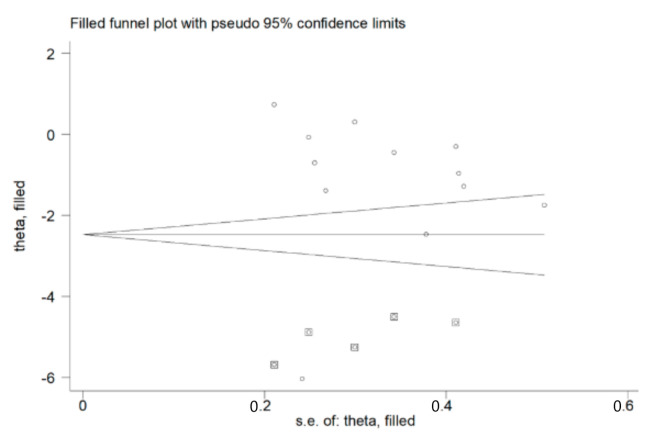
Funnel plot of studies investigating healthy controls and patients with COPD after trimming and filling. Dummy studies and genuine studies are represented by enclosed circles and free circles, respectively.

The analysis of the 12 remaining studies showed a lack of publication bias (Begg’s test, *p* = 0.19; Egger’s test, *p* = 0.95). The trim-and-fill method found five potential missing studies to add on the left side of the funnel plot to ensure the symmetry (Figure 5), with a resulting adjusted SMD of −2.32; 95% CI = −3.57 to −1.07, *p* < 0.001.

**Figure 6:**
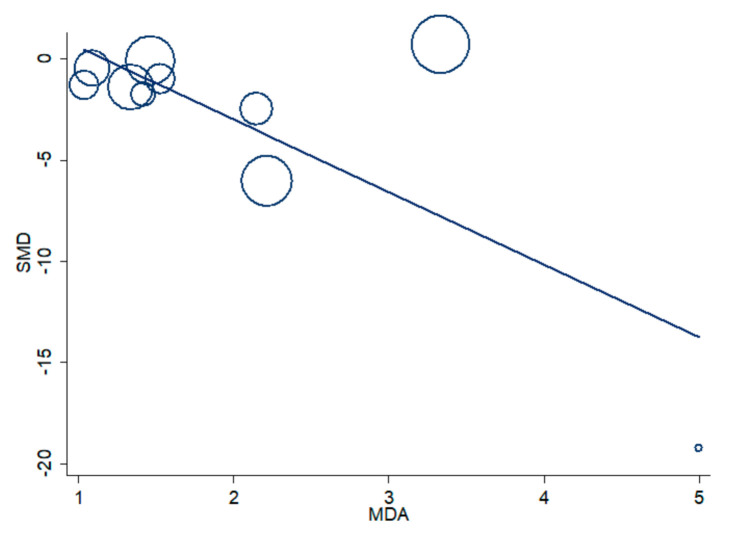
Meta-regression analysis of malondialdehyde serum concentration and SMD.

The age, gender, body mass index (BMI), forced expiratory volume at 1 s (FEV1), FEV1/forced vital capacity (FVC), malondialdehyde (MDA), biological matrix (RBCs/WB), continent where the study was conducted (Europe, Africa or Asia), publication year and guidelines used for COPD diagnosis were investigated as potential contributors to the between-study variance in meta-regression analysis. The MDA levels were significantly associated with the SMD (t = −3.40, *p* = 0.009, Figure 6).

**Figure 7:**
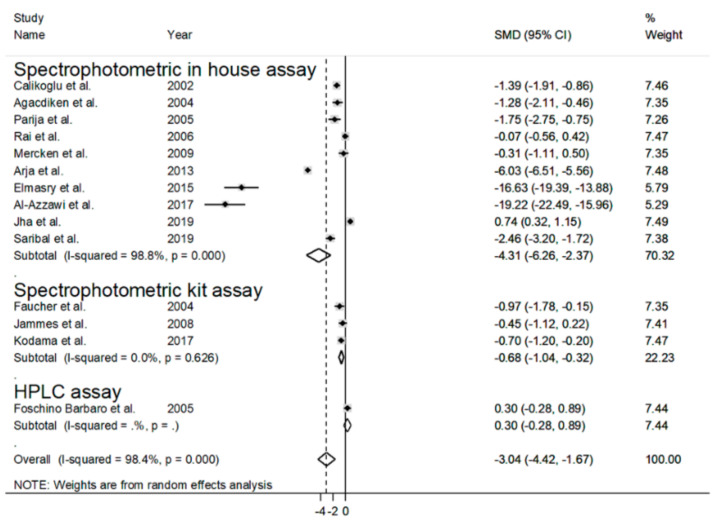
Forest plot of studies examining blood reduced GSH and COPD according to assay method used to detect GSH.

By contrast, no significant associations were observed with age (t = −1.71, *p* = 0.12), gender (t  =  0.52, *p*  =  0.61), BMI (t  =  −1.13, *p*  =  0.34), FEV1 (t  =  0.64, *p*  =  0.55), FEV1/FVC (t  =  −0.70, *p*  =  0.51), biological matrix (t = −1.60, *p* = 0.14), continent (t  =  −1.45, *p*  =  0.18), publication year (t  =  −1.54, *p*  =  0.15) or type of guidelines (t  =  −1.45, *p*  =  0.17). Methodological factors, such as the use of an in-house spectrophotometric assay vs. commercial spectrophotometric kit, were also explored as potential contributors to the heterogeneity (Figure 7). The SMD values in studies using in-house assays (SMD = −4.31, 95% = −6.26 to −2.37, *p* < 0.001; I^2^ = 98.8%, *p* < 0.001) were not significantly different from those in studies using commercial ones (SMD = −0.68, 95% CI = −1.04 to −0.32, *p* < 0.001; I^2^ = 0.0%, *p* < 0.63; t = 0.89, *p* = 0.39).

### 3.3. Blood Total GSH (tGSH) and COPD

**Figure 8:**
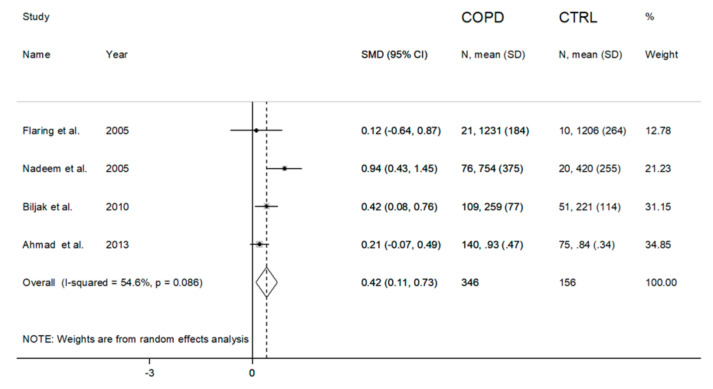
Forest plot of studies examining blood total GSH and COPD.

Blood tGSH concentrations were significantly higher in COPD patients vs. controls in three studies [33,38,43], while no significant between-group differences were observed in one study [32]. A forest plot of blood tGSH concentrations in COPD patients vs. healthy subjects is presented in Figure 8.

**Figure 9:**
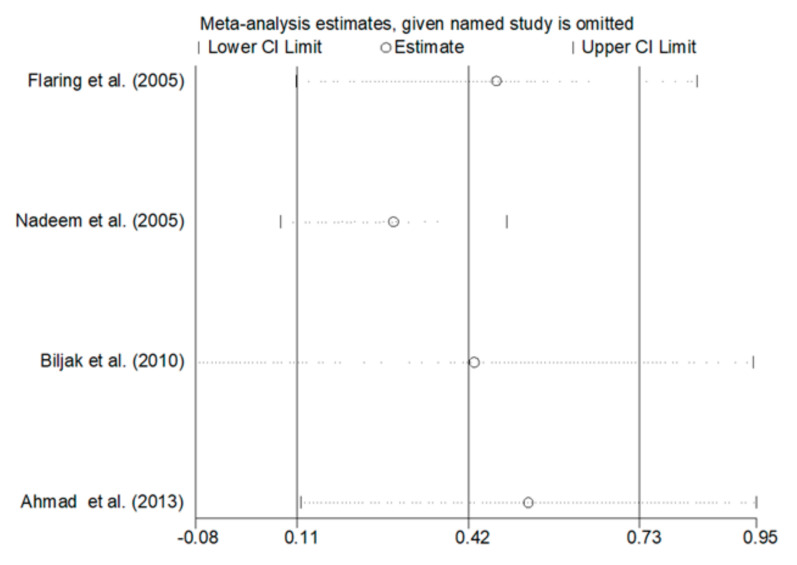
Sensitivity analysis of the association between blood total GSH and COPD. The influence of individual studies on the overall standardized mean difference (SMD) is shown. The middle vertical axis indicates the overall SMD, and the two vertical axes indicate the 95% confidence intervals (CI). Hollow circles represent the pooled SMD when the remaining study was omitted from the meta-analysis. Two ends of each broken line represent 95% CIs.

A large heterogeneity among the studies was observed (I^2^  =  54.6%, *p*  =  0.086); therefore, a random-effects model was used to compute the overall effect. The pooled SMD showed that tGSH concentrations were significantly higher in COPD patients than controls (SMD = 0.42, 95% CI = 0.11 to 0.73; *p*  = 0.009). As shown in Figure 9, the effect size did not substantially change following the removal of each single case-control study at a time (the effect size ranged between 0.28 and 0.53). An analysis of the publication bias and meta-regression analysis were not performed because of the limited number of studies.

## 4. Discussion

Although the presence of systemic oxidative stress in COPD patients is generally recognized, less clear is the evidence regarding possible alterations in the blood concentrations of GSH, a key non-enzymatic antioxidant, in this group. Methodological factors related to blood GSH measurement, such as the blood matrix used (WB, RBCs or plasma/serum), the form of GSH measured (reduced or total), the baseline concentrations in different blood matrices, the differences in the handling and pre-treatment of samples and the analytical steps, could account for the contrasting results of published studies. We sought to address these issues by selecting observational studies that specifically measured GSH in RBCs/WB, carefully distinguishing those that measured the reduced form from those that assessed the total form. We excluded primary studies assessing GSH in the plasma/serum, as the much lower basal concentrations of GSH in such matrices, 2–4 µmol/L in the plasma vs. 0.6–3.6 mmol/L in RBCs, were more likely to be affected by potential differences in the sample handling and/or analytical procedures between the studies [21,50]. Similarly, only primary case-control studies were selected, on the basis that any pre-analytical and analytical error would similarly affect both cases and controls. Finally, the SMD was used to make uniform the different scales of measurement used to express GSH concentrations and to minimize the potential effects of improper pre-analytical and/or analytical treatment. Eighteen primary studies, 14 reporting rGSH and four tGSH, fulfilled the inclusion criteria and were further analyzed. Pooled analysis showed that COPD patients have significantly lower rGSH, and higher tGSH, concentrations when compared to healthy controls. The lack of a significant reduction in tGSH concentrations in COPD patients suggests the increased oxidation, rather than impaired synthesis, of this thiol. Thus, taken together, these data suggest a downregulation of blood GSH concentrations in COPD resulting from a systemic state of oxidative stress. Meta-regression analysis showed that the observed alterations in GSH concentrations were not associated with age, gender, BMI, FEV1, FEV1/FVC, continent where the study was conducted, publication year, type of guidelines or biological matrix. Similarly, the pooled SMD (size and direction) was not significantly affected by whether an in-house spectrophotometric assay or commercial spectrophotometric kits were used (t = 0.89, *p* = 0.39). However, the extreme heterogeneity observed in studies using home-made methods (I^2^ = 98.8%, *p* < 0.001), which do not have to comply with the standardization requirements for commercial kits, highlights, once again, the importance of methodological factors as a source of variability. Thus, the extreme (I^2^  =  98.4%, *p*  <  0.001) and large (I^2^  =  54.6%, *p*  =  0.086) heterogeneity observed among the studies estimating, respectively, rGSH and tGSH might also be explained, at least partly, by analytical factors. In most of the retrieved articles, in fact, the sample processing involved the lysis of RBCs and removal of proteins by an acidic step using different reagents such as perchloric acid, trichloroacetic acid, metaphosphoric acid or sulfosalicylic acid. However, it is well-documented that the release of hemoglobin following acidic treatment induces the oxidation of a fraction of GSH, with the associated formation of glutathione disulfide, the occurrence of which is often an artifact [51,52,53]. Moreover, the detection of GSH is frequently performed using Ellman’s reagent, which is not a specific reagent for GSH, as it can react with any free sulfhydryls in solution [54]. Even blood withdrawal may lead to inconsistencies, as the partial oxidation of GSH resulting from an incorrect sample collection technique (e.g., butterfly needle/vacutainer vs. syringe) might occur [55]. Therefore, these methodological issues may affect the variability in GSH concentrations in individual studies. On the other hand, the heterogeneity could also be linked to specific subject characteristics, e.g., smoking and COPD stage. The association observed between the pooled GSH SMD and MDA concentrations in the meta-regression analysis (t = −3.40, *p* = 0.009, Figure 6) supports this proposition, as an increase in plasma MDA concentrations is common in patients with advanced COPD [56,57]. As shown in Figure 3 and Figure 4, the heterogeneity could also be explained by the distortive effect of the studies measuring rGSH by Elmasry et al. [45] and Al-Azzawy et al. [47]. However, their removal did not substantially reduce the magnitude of the heterogeneity (I^2^  =  98.0%, *p* < 0.001), although a reduction in effect size was observed (SMD = −1.20, 95% CI = −2.42 to 0.030, *p* = 0.056). No publication bias was found using Begg’s (*p* = 0.19) and Egger’s (*p* = 0.95) tests following the removal of these studies. However, the trim-and-fill method found five potential missing studies to add on the left side of the funnel plot to ensure its symmetry (Figure 5), yielding an adjusted SMD of −2.32; 95% CI = −3.57 to −1.07, *p* < 0.001. Although publication bias analysis was not performed due to the reduced number of articles available, similar results were obtained when removing studies measuring tGSH (Figure 9). Taken together, these analyses rule out significant issues related to the meta-analysis design as a source of heterogeneity.

## 5. Conclusions

To our knowledge, this is the first meta-analysis exploring the association between blood GSH concentrations and COPD. It is important to highlight that our analysis was limited to observational studies investigating GSH concentrations in RBCs/WB, biological matrices that are less likely to be affected by analytical shortcomings. The substantial heterogeneity among the included studies can still be explained by methodological factors, e.g., sample collection and handling, as well as specific patient characteristics, rather than issues related per se to the meta-analysis design. Thus, although a random-effects model was used to calculate the pooled standardized mean difference and the number of articles identified for the meta-analysis were limited, the significant reduction in blood rGSH concentrations observed in COPD patients when compared to healthy controls represents a robust finding that supports the presence of an impaired antioxidant defense mechanism in COPD.

## Figures and Tables

**Table 1:** Summary of the studies on chronic obstructive pulmonary disease (COPD) vs. controls included in the meta-analysis. * Mean and standard deviation (SD) were estimated from formulas using the median and range as described by Wan et al. [4]. NOS: Newcastle–Ottawa quality assessment scale for case–control studies. NR: not reported; Red: reduced; Tot: total. RBC: red blood cell; WB: whole blood.

						Control Group				COPD Group			
First Author, Year, Country	Diagnosis	NOS (Stars)	Matrix	GSH Form	Measurement Units	*n*	Age (Years)	Gender (M/F)	GSH Mean ± SD	*n*	Age (Years)	Gender (M/F)	GSH Mean ± SD
Calikoglu et al. 2002, Turkey	BTS	8	RBC	Red	μmol/gHb	30	62.56	15/15	4.75 ± 0.64	41	58.21	20/21	3.49 ± 1.06
Agacdiken et al. 2004, Turkey	NR	8	WB	Red	mg/dL	10	49	10/0	22.4 ± 16.6	21	63	21/0	8.37 ± 7
Faucher et al. 2004, France	NR	6	RBC	Red	nmol/mL	8	59	7/1	1511 ± 461	30	66	29/1	1006 ± 537
Foschino Barbaro et al. 2005, Italy	GOLD	6	RBC	Red	µmol/L	15	61	15/0	741 ± 101	45	62	45/0	769 ± 89
Flaring et al. 2005, Sweden	NR	6	WB	Tot	µmol/L	10	42	3/7	1206 ± 264 *	21	75	NR	1231 ± 184 *
Nadeem et al. 2005, India	GOLD	8	WB	Tot	mmol/L	20	NR	20/0	0.420 ± 0.057	76	NR	76/0	0.754 ± 0.043
Parija et al. 2005, India	NR	8	WB	Red	mg/gHb	11	55	9/2	1.79 ± 0.24	11	57	10/1	1.37 ± 0.24
Rai et al. 2006, India	NR	6	WB	Red	mg/dL	50	22–55	NR	30.6 ± 5.75	24	35–65	NR	30.2 ± 5.39
Jammes et al. 2008, Senegal	ATS/ERS	6	RBC	Red	nmol/mL	18	48	13/5	1953 ± 1107	17	53	13/4	1565 ± 495
Mercken et al. 2009, Netherlands	GOLD	9	RBC	Red	µmol/gHb	10	56	4/6	4.30 ± 1.24	15	57	10/5	3.82 ± 1.75
Biljak et al. 2010, Croatia	GOLD	6	RBC	Tot	µmol/L	51	52	21/30	221 ± 114 *	109	71	82/27	259 ± 77 *
Ahmad et al. 2013, India	GOLD	6	WB	Tot	mmol/L	75	42	53/22	0.84 ± 0.34	140	45	111/29	0.93 ± 0.47
Arja et al. 2013, India	GOLD	6	RBC	Red	nmol/mL	150	61	150/0	4.02 ± 0.17	236	63	236/0	2.88 ± 0.20
Elmasry et al. 2015, Egypt	GOLD	6	WB	Red	mg/dL	40	54	31/9	23.3 ± 0.6	34	55	27/7	8.9 ± 1.1
Kodama et al. 2017, Japan	GOLD	6	WB	Red	µmol/L	28	69	19/9	892 ± 122	39	73	34/5	742 ± 262
Al-Azzawi et al. 2017, India	ATS/ERS	6	WB	Red	µg/mL	40	45	28/12	41.53 ± 1.54	30	65	21/9	13.8 ± 1.3
Jha et al. 2019, India	GOLD	6	RBC	Red	µmol/gHb	40	55	31/9	8.3 ± 1.1	60	59	36/24	9.2 ± 1.3
Saribal et al. 2019, Turkey	NR	7	RBC	Red	mg/dL	25	NR	25/0	25.7 ± 2.1	25	NR	25/0	16.1 ± 5.1
